# Optimized binding of substituted quinoline ALLINIs within the HIV-1 integrase oligomer

**DOI:** 10.1016/j.jbc.2021.100363

**Published:** 2021-02-02

**Authors:** Jian Sun, Krunal Patel, Jared Hume, Julie A. Pigza, Matthew G. Donahue, Jacques J. Kessl

**Affiliations:** 1 Department of Chemistry and Biochemistry, University of Southern Mississippi, Hattiesburg, Mississippi, USA; 2 Center for Molecular and Cellular Biosciences, University of Southern Mississippi, Hattiesburg, Mississippi, USA

**Keywords:** human immunodeficiency virus, integrase, antiviral agent, drug design, protein–protein interaction, oligomerization, quinoline, ALLINI, ALLINI, allosteric IN inhibitors, CCD, catalytic core domain, CTD, C-terminal domain, DMSAO, dimethyl sulfoxide, HIV-1, human immunodeficiency virus type 1, HTRF, homogeneous time resolved fluorescence, I N, integrase, NTD, N-terminal domain, RNP, ribonucleoprotein complex, vDNA, viral cDNA

## Abstract

During the integration step, human immunodeficiency virus type 1 integrase (IN) interacts with viral DNA and the cellular cofactor LEDGF/p75 to effectively integrate the reverse transcript into the host chromatin. Allosteric human immunodeficiency virus type 1 integrase inhibitors (ALLINIs) are a new class of antiviral agents that bind at the dimer interface of the IN catalytic core domain and occupy the binding site of LEDGF/p75. While originally designed to block IN-LEDGF/p75 interactions during viral integration, several of these compounds have been shown to also severely impact viral maturation through an IN multimerization mechanism. In this study, we tested the hypothesis that these dual properties of ALLINIs could be decoupled toward late stage viral replication effects by generating additional contact points between the bound ALLINI and a third subunit of IN. By sequential derivatization at position 7 of a quinoline-based ALLINI scaffold, we show that IN multimerization properties are enhanced by optimizing hydrophobic interactions between the compound and the C-terminal domain of the third IN subunit. These features not only improve the overall antiviral potencies of these compounds but also significantly shift the ALLINIs selectivity toward the viral maturation stage. Thus, we demonstrate that to fully maximize the potency of ALLINIs, the interactions between the inhibitor and all three IN subunits need to be simultaneously optimized.

The integrase (IN) protein of human immunodeficiency virus type 1 (HIV-1) catalyzes the insertion of viral cDNA (vDNA) into the host chromosome. This catalytic activity of IN is essential for the early stage of the viral replication and has been developed as therapeutic target. Four FDA-approved inhibitors (raltegravir, elvitegravir, dolutegravir, and bictegravir) that bind at the catalytic site are now commonly used clinically to treat HIV-1 infected individuals. The HIV-1 IN is comprised of three structurally distinct domains: the N-terminal domain (NTD), the catalytic core domain (CCD), and the C-terminal domain (CTD) (1, 2). During integration, these three domains work in conjunction to form a stable tetrameric structure where vDNA and host chromosomal DNA are bound to IN (3, 4, 5, 6). Efficient integration of the HIV-1 genome in infected cells also requires the interaction between IN and the cellular chromatin-associated protein LEDGF/p75 which acts as a bimodal tether to link the IN-vDNA complex to active genes (7, 8, 9, 10, 11, 12). The LEDGF/p75 association with the host chromatin is mediated through its N-terminal segment which selectively recognizes the H3K36me3 histone mark (13). LEDGF/p75 also binds the IN tetramer through its C-terminal integrase binding domain by inserting a small loop into a v-shaped cavity located at the IN CCD dimer interface (10, 14). Allosteric IN inhibitors (ALLINIs) (15, 16, 17, 18, 19), which are also known as LEDGINs (LEDGF/p75 inhibitors) (20), noncatalytic site integrase inhibitors (21), or IN-LEDGF allosteric inhibitors (22), selectively bind at the IN CCD dimer interface at the LEDGF/p75 binding pocket, away from the IN catalytic site and potently inhibit HIV-1 replication in cell culture. While originally designed to block IN-LEDGF/p75 interactions, these compounds have been shown to also impact IN functions by inducing aberrant IN multimerization (15, 21, 23).

Studies on the antiviral mechanism of action of ALLINIs have revealed that these compounds not only inhibit integration but also unexpectedly impede the late stages of HIV-1 replication. Closer observations have shown that the binding of these compounds significantly interfere with proper virus particle maturation and yield noninfectious particles. A hallmark of this drug-induced defect is the mislocalization of ribonucleoprotein complexes (RNPs) to an eccentric position between the empty capsid (CA) core and the particle membrane in mature virions, whereas normal virions contain RNPs within the CA core. Thus, IN plays a critical noncatalytic role during viral particle maturation that can be probed or targeted using ALLINIs. The use of these intriguing compounds has emerged as a promising, complementary approach to active site-based inhibitors of HIV-1 IN (24). Through computer simulations (25), crystallography (26), and biochemical experiments (27, 28), it has been previously suggested that in addition to binding at the IN CCD dimer interface, some ALLINIs could also bridge with the CTD of a third IN subunit (Fig. 1), a feature that could be important for the development of the antiviral potency of this class of inhibitors. To further probe these interactions, we have synthesized and tested a series of quinoline derivatives aiming to maximize the contacts with all three subunits (Fig. 2).

**Figure 1:**
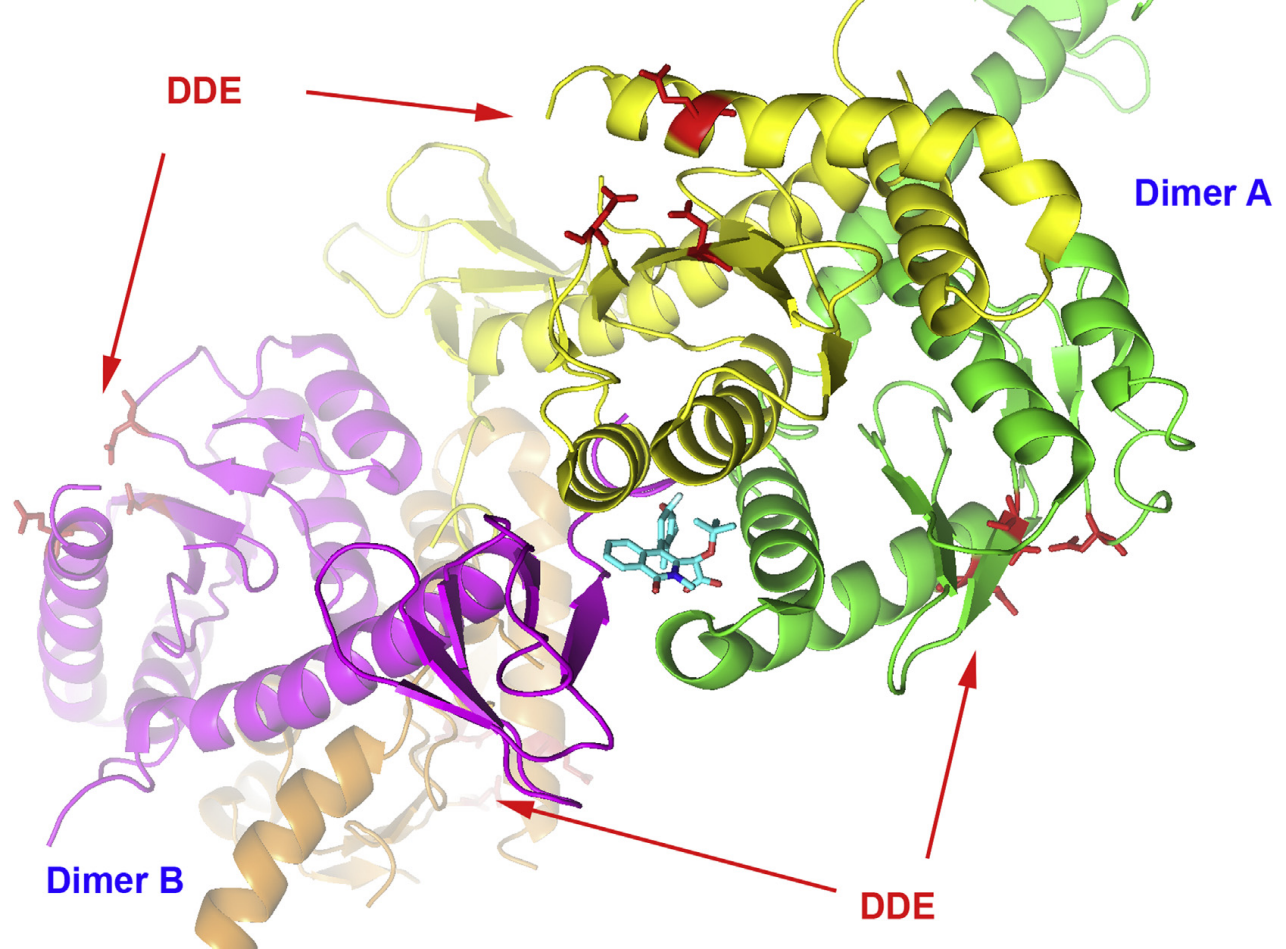
**ALLINI binding to HIV-1 IN tetramer.** The ALLINI compound (*cyan*) binds at the dimeric interface composed of catalytic core domains of subunits *yellow* and *green* (dimer A) and interacts with the C-terminal domain *magenta* of a third subunit (dimer B, subunits *magenta* and *orange*). The position of the catalytic triad DDE (D64, D116, and E152) is indicated. View generated using the structure 5HOT. ALLINI, allosteric IN inhibitor; HIV-1, human immunodeficiency virus type 1; IN, integrase.

**Figure 2:**
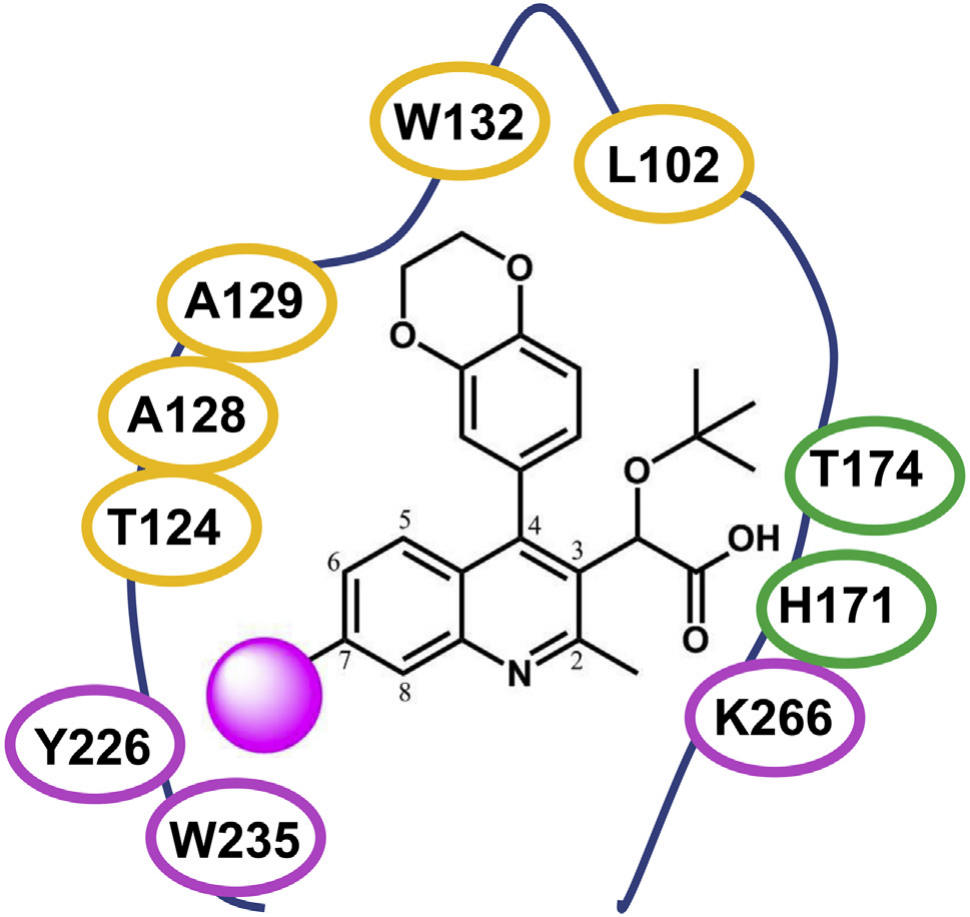
**Binding pocket with compound NGJ9002.** Interaction of ALLINI compound NGJ9002 (with position numbers) with residues of the three HIV-1 IN subunits (*yellow*, *green*, and *magenta*). The *magenta ball* represents the position derivatized. ALLINI, allosteric IN inhibitor; HIV-1, human immunodeficiency virus type 1; IN, integrase.

## Results

### Contribution of the quinoline position 7 to the drug-induced IN multimerization

The compounds belonging to the ALLINI class share several common structural features including a central aromatic scaffold (quinoline, isoquinoline, pyrimidine, indole, or others) which have been the subject of several substitution studies that have been able to modulate the potency of these molecules. For example, early works have shown that the 3-α-tert-butoxy acetic acid side-chain (quinoline scaffold position numbers in Fig. 2) is optimal for IN anchoring (15, 16, 20) because it provides several hydrogen bond interactions with T174 and H171 residues that mimic the LEDGF/p75 binding pattern. More recent derivatization efforts have focused on the position 4 (19, 29). We and others have found that large aromatic substitutions at this site greatly enhance multimerization properties of the compounds by maximizing interaction with the hydrophobic pocket (19, 29) including residues W132 and L102 (Fig. 2). In the present work, to further understand and quantify the specific contribution of each feature of these inhibitors toward multimerization, we have kept constant the 3-α-tert-butoxy acetic acid side-chain and our previously optimized position 4 with a 1,4-benzo dioxane group (19). Here, we have selected to further derivatize our quinoline scaffold at position 7 to test if an increase of compound interaction with the residues Y226 and W235 of the third subunit (magenta in Figs. 1 and 2) would enhance IN multimerization properties.

Preliminary examination of the available ALLINI-bound IN tetramer (26) (Fig. 1) revealed that size constrains and residues polarity would accommodate phenyl derivatives at position 7. The double substitution at both the 4 and 7 positions was obtained by a sequential Suzuki cross-coupling strategy where selected boronic acid analogs were added to halogens on the quinoline scaffold using a palladium catalyst under basic conditions (detailed chemical synthesis procedures and complete compounds characterization are included in the Supporting Information section). The α-tert-butoxy-acetic acid was then recovered through saponification (Fig. S1). To test our hypothesis, we first obtained KHP1095 (Table 1) which added a simple phenyl group at position 7. Testing in dose–response measurements using the previously described (15, 30) homogeneous time resolved fluorescence (HTRF)-based IN multimerization assay showed an improvement in EC_50_ value compared with our published (19) unsubstituted compound NGJ9002 from 0.11 to 0.07 μM. Additional observations of the available ALLINI-bound IN tetramer X-ray structure (26) suggested that compound binding could be further improved by additional hydrophobic interactions with W235 of the third subunit (magenta in Figs. 1 and 2). Interestingly, this effect could be achieved by adding aliphatic substituents on ortho position of the phenyl ring. Thus, six additional ortho phenyl–substituted quinoline analogs were then generated and tested for *in vitro* IN multimerization (Table 1). Among those, three compounds displayed better EC_50_ values than KPH1095, with JDH2110 having the lowest multimerization EC_50_ value at 0.04 μM. Finally, to further explore the properties of this series, we synthesized and tested both the meta (JDH2102) and the para (KHP1123) variants of JDH2110 (Table 2). While the para substitution (KHP1123) displayed a multimerization EC_50_ of 0.04 μM like the ortho (JDH2110), we observed a notably less potent value for the meta variant (JDH2102) with 0.52 μM.

**Table 1:** *In vitro* IN multimerization EC_50_ activities of substituted quinoline-based ALLINIs


Compound	R	EC_50_ (μM)
NGJ-9002		0.11 ± 0.01
KHP-1095	H	0.07 ± 0.01
KHP-1056	Cl	0.11 ± 0.01
KHP-1050	CH_3_	0.06 ± 0.01
KHP-1091	CF_3_	0.14 ± 0.01
JDH-2110	OCH_3_	0.04 ± 0.01
KHP-1092	OCF_3_	0.12 ± 0.01
KHP-1099	OC_2_H_5_	0.06 ± 0.01

ALLINI, allosteric IN inhibitor; IN, integrase.

Average values with SD are shown for three independent experiments.

**Table 2:** *In vitro* IN multimerization EC_50_ activities of substituted quinoline-based ALLINIs

KHP-1123	JDH-2102	JDH-2110
Compound	OCH_3_ position	EC_50_ (μM)
KHP-1123	para	0.04 ± 0.01
JDH-2102	meta	0.52 ± 0.01
JDH-2110	ortho	0.04 ± 0.01

ALLINI, allosteric IN inhibitor; IN, integrase.

Average values with SD are shown for three independent experiments.

### Contribution of the IN-CTD to the drug-induced IN multimer complex

**Figure 3:**
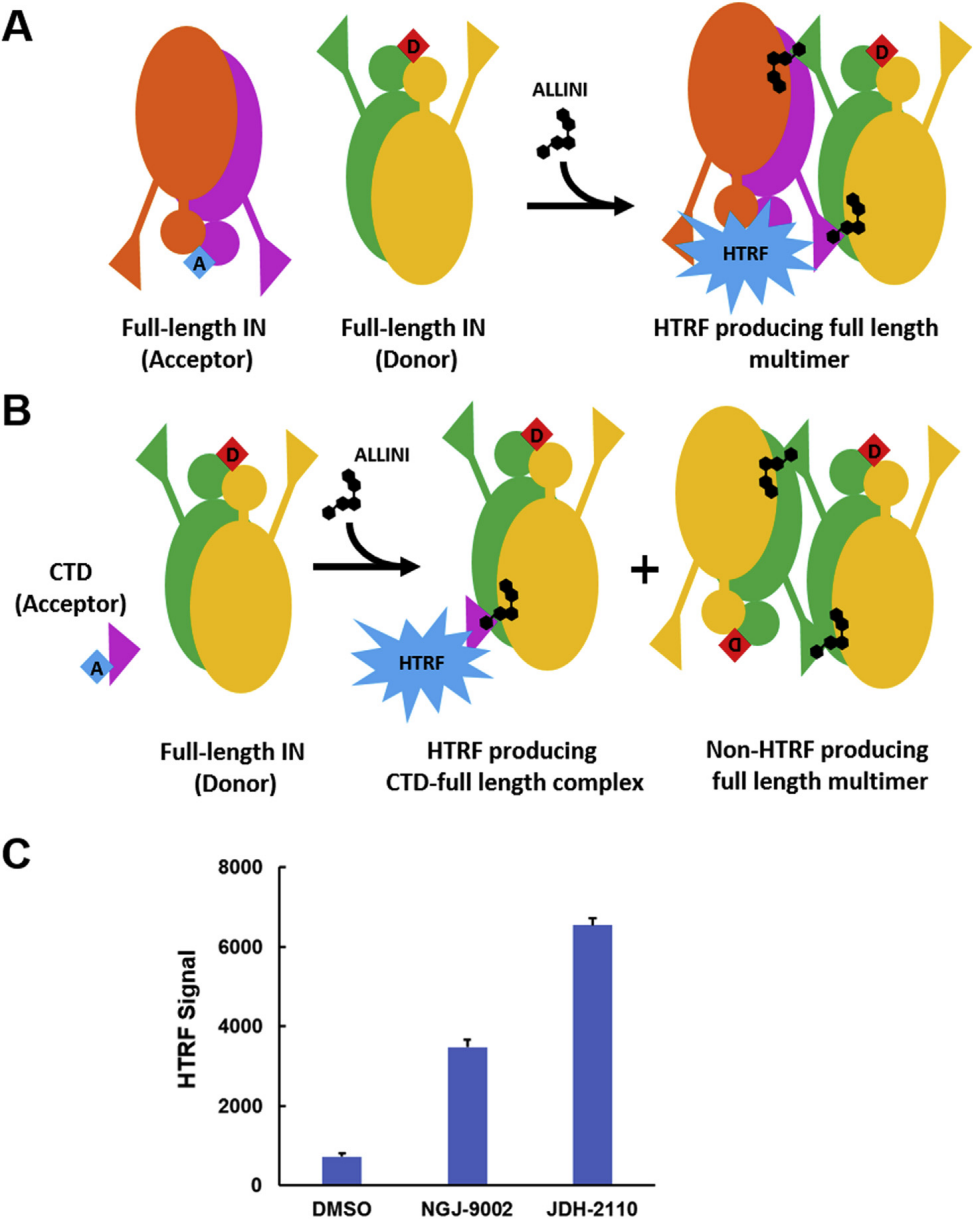
**C-terminal domain (CTD) contribution to the ALLINI-induced IN multimer formation.***A*, schematic of the standard HTRF-based IN multimerization assay. The addition of a potent ALLINI compound (*black molecule*) to a mixture of full-length IN proteins interacting with antibodies labeled with either donor (*red D label*) or acceptor (*blue A label*) fluorophores induces the formation of a HTRF producing IN multimer. *B*, schematic of the HTRF-based CTD binding assay: The addition of a potent ALLINI compound (*black molecule*) to a mixture of full-length IN and CTD domain proteins interacting with the same fluorophore-labeled antibodies induces the formation of a HTRF producing CTD full-length IN complex. *C*, HTRF signal intensities recorded after the addition of 200 nM of ALLINI to a mixture of Flag tagged full-length IN and His-tagged CTD domain proteins as described in the Methods section. ALLINI, allosteric IN inhibitors; HTRF, homogeneous time resolved fluorescence; IN, integrase.

To detect in isolation the specific contribution of the IN-CTD to the ALLINI-induced IN multimer complex formation, we have modified our published HTRF-based assay (Fig. 3, *A* and *B*). In this new analytical assay, anti-His6-XL665 and anti-FLAG-EuCryptate antibodies allow the detection of fluorescence energy transfer only when FLAG-tagged full-length IN protein tightly interacts with a His-tagged IN-CTD construct. Mixing of the proteins and without compound addition generated a small HTRF signal (Fig. 3*C*, dimethyl sulfoxide [DMSO]) indicating a weak interaction between the full-length IN and the IN-CTD construct. Strikingly, the addition of ALLINI to the assay promoted a significant increase of HTRF signals proportional to the IN multimerization properties of the compound. The data in Figure 3*C* show that JDH2110 generated higher HTRF than NGJ9002, suggesting additional contact points with the IN-CTD for this compound. On note, while the ALLINI-induced multimerization of the FLAG tagged full-length IN protein is likely to occur in the assay, it does not contribute to the recorded signal (Fig. 3*B*).

### Molecular modeling of the inhibitor binding to IN

#### Docking of the benzo-dioxane quinoline scaffold into the IN tetrameric structure

While a large number of crystal structures showing ALLINIs interacting with the LEDGF-integrase binding domain binding pocket exist (15, 16, 17, 18, 20, 22, 31, 32), they are limited to the IN CCD dimer interface (subunits green and yellow in Figs. 1 and 2) and do not describe the potential ALLINI interactions with the third subunit (magenta in Figs. 1 and 2). Thus, an energy-minimized conformation of NGJ9002 was docked into the ALLINI binding pocket of the only available X-ray structure (pdb: 5HOT) obtained with full-length IN of the ALLINI-induced IN tetrameric assembly (26). The isoquinoline GSK1264 co-crystallized in the structure was used to guide the initial positioning of NGJ9002. Specifically, both 3-α-tert-butoxy acetic acid side-chains were overlaid so hydrogen bonds between the compound’s carboxyl group, and the residues H171/T174 (subunit green in Fig. 2) were conserved. On note, while our compounds were synthesized as racemic mixtures, we selected to use the same stereoisomeric *S*-configuration for the 3-α-tert-butoxy acetic acid side-chain of our docked quinolines. The isoquinoline and quinoline core scaffolds of GSK1264 and NGJ9002, respectively, were likewise overlaid to maximize interactions with residues T125/A128/A129 (subunit yellow in Fig. 2). To position and angle the benzo-dioxane group in position 4, we selected to use the orientation of the chroman group from the X-ray BID-IN CCD complex structure (pdb: 4ID1) (31), as this group more closely resemble our substitution. Using a CHARMM-based forcefield setting, a sequence of dynamic cascade runs that included minimization steps were applied to the structure with the docked NGJ9002 in which the lowest energy conformation was selected (details in Methods section). An overlaid view of the starting isoquinoline GSK1264 X-ray structure (26) (pdb: 5HOT) with the calculated NGJ9002 model showed only minor variation of the binding pocket (Data not shown).

#### Binding energy calculation of the 7-substitued quinolines

**Figure 4:**
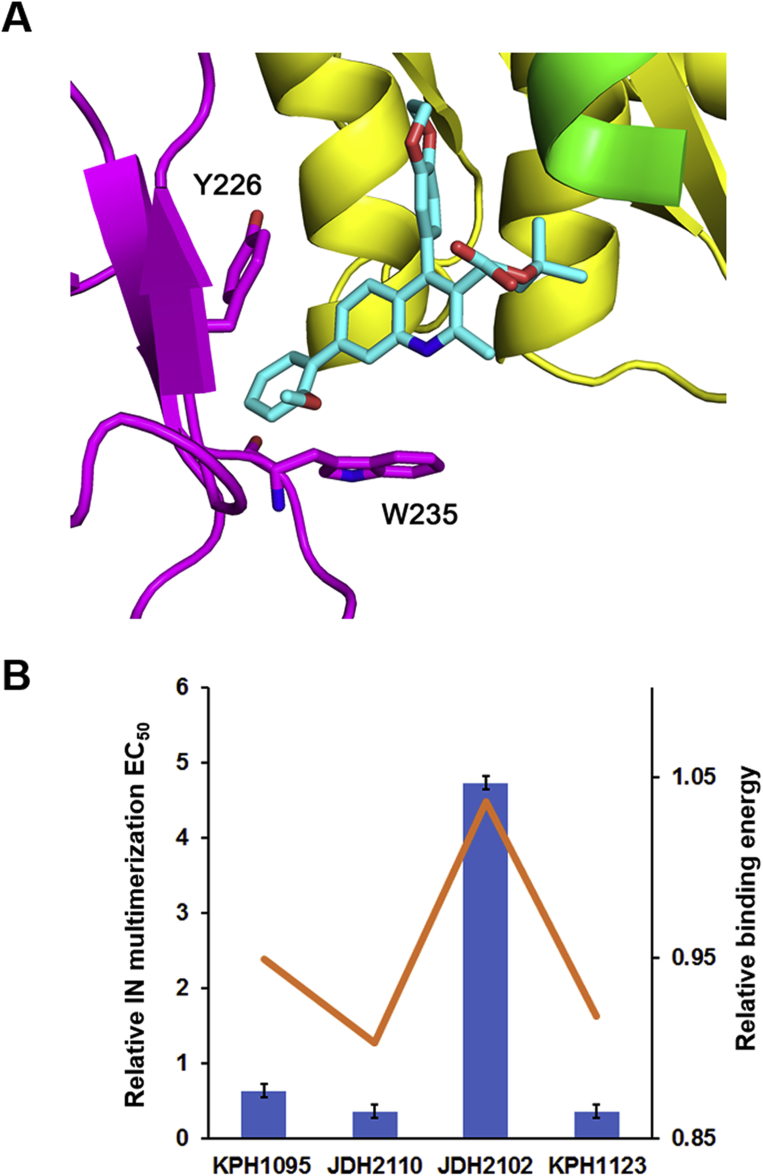
**Molecular modelization and correlation with experimental EC50s.***A*, energy minimized binding model of compound JDH2110. The ALLINI compound (*cyan*) interacts with the three HIV-1 IN subunits (*yellow*, *green*, and *magenta*). Amino acids Y226 and W235 from the third subunit CTD (*magenta*) are shown. *B*, calculated binding energies corelate with experimentally measured IN multimerization EC_50_. For each compound, the fold increase in EC_50_ values relative to the unsubstituted compound (NGJ9002) for IN multimerization is shown with *blue bars* (average values of three independent experiments and corresponding SDs are shown). The *orange line* shows the fold increase in calculated binding energy relative to the same unsubstituted compound (NGJ9002). ALLINI, allosteric IN inhibitors; CTD, C-terminal domain; HIV-1, human immunodeficiency virus type 1; IN, integrase.

The calculated NGJ9002 structure from above was used as starting point to generate binding models for compounds KPH1095, JDH2110, JDH2102, and KPH1123. For this series, additional flexibility features were incorporated in the CTD section of the model (magenta in Figs. 1 and 2) to accommodate the steric hindrance of the substituted benzyl group added on position 7 of the quinoline scaffold. A representative display of the energy minimized binding model of JDH2110 is shown on Figure 4*A*. In this model, the ortho-substituted benzyl group maximize pi-pi interactions with aromatic residues Y226 and W235 of the third subunit (magenta in Fig. 4*A*). To further validate our modelization approach, the calculated binding energy for each compound was extracted from the models and compared with the experimentally measured multimerization EC_50_ values (Fig. 4*B*). The improvement in calculated binding energies significantly correlated with the decrease in multimerization EC_50_ values for each substituted benzyl group derivative as shown in Figure 4*B*. A larger negative effect on the multimerization properties was observed for meta-substitution (JDH2102) for which the calculation predicted smaller variation.

### Antiviral activities of selected inhibitors

**Figure 5:**
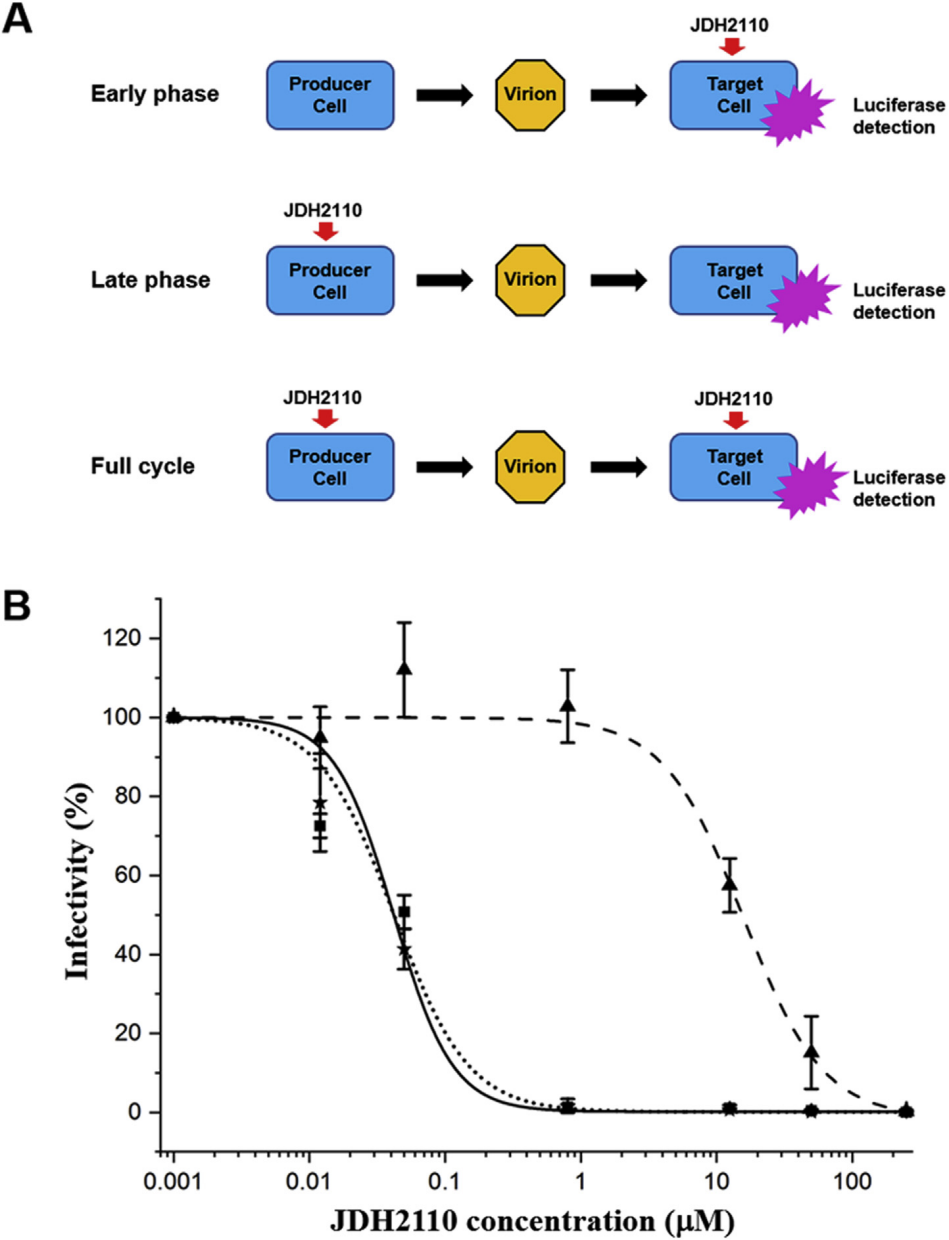
**JDH2110 is more potent during the late phase of HIV-1 replication.***A*, schematic of the of the antiviral methodologies used to assess JDH2110 potency at early and/or late phase of the HIV-1 viral cycle. *B*, dose–response curves under early (*triangles*, *dashed line*), late (*squares*, *dotted line*), and full cycle (*stars*, *solid line*) conditions of drug treatment. The error bars represent the variation obtained from three independent experiments. HIV-1, human immunodeficiency virus type 1.

To confirm the potency of our best quinoline analog, JDH2110, we have surveyed their effects on HIV-1 replication in cell culture. Several studies (16, 17, 31) have demonstrated that ALLINIs do not only affect the integration step but also significantly impair the infectivity of HIV-1 progeny virions without impacting viral particles production. Thus, to dissect the inhibition mechanism of our compounds, we have examined their effects at both early and late stages of HIV-1 replication by timing the inhibitor addition to either target or producer cells (Fig. 5*A*). For early stage experiments, viral infectivity of target cells was measured in the presence of increasing concentrations of inhibitors. For late stage, producer cells were transfected in the presence of increasing concentrations of inhibitors, cell-free virions were harvested at 48 h, and the amounts of viral particles produced were measured by p24 concentration essay. Subsequently, the infectivity of these progeny virions was examined without any additional inhibitor being added to the target cells. Under these conditions, only a negligible amount of the compound was transferred from the producer cells to the target cells based on the volumes used for the infections. For full viral cycle experiments, the same inhibitor concentrations were added in the producer cells and the target cells (31). Figure 5*B* shows a representative dose–response plotting of the three measurement methods (early phase, late phase and full cycle) obtained with JDH2110.

To specifically weigh the contribution effects on position 7 of our quinoline scaffold, we have selected to compare side by side the compounds NGJ9002, KPH1095, and JDH2110. The antiviral potency of our quinoline series was clearly impacted by the nature of the substitution at position 7. The values obtained for the full viral cycle experiments (Table 3) correlated significantly with the *in vitro* IN multimerization EC_50_ measurements (Table 1). When added to the producer cells, these quinoline compounds inhibited the viral infectivity of the progeny virions with IC_50_ which closely correlated with the values obtained in full replication cycle. In contrast, these inhibitors were significantly less effective when added to the target cells during infection (Table 3 and Fig. 5). Because the overall potency of our substituted quinoline series was observed at the late stage, we defined a PSI that measures the ratio between experimental IC_50_ obtained at early and late phases of viral replication. The PSI value variations obtained with the compounds NGJ9002, KPH1095, and JDH2110 (Table 3) point to the significant contribution of the substitution at position 7 of our quinoline scaffold. By adding a phenyl group, the PSI increased 4-fold (NGJ9002 *versus* KPH1095) while adding an ortho-substituted methoxy-phenyl group increased the PSI by 7-fold (NGJ9002 *versus* JDH2110). As previously reported with other quinoline scaffold-based ALLINIs (22, 29, 32), no measurable cytotoxicity was detected in the concentration ranges tested (Table 3).

**Table 3:** CC_50_ toxicity values, IC_50_ values for the indicated antiviral assay conditions, and phase specificity index

NGJ-9002		KHP-1095		JDH-2110	
Compound	CC_50_ (μM)	Full IC_50_ (μM)	Early IC_50_ (μM)	Late IC_50_ (μM)	PSI
NGJ-9002	>100	0.83 ± 0.05	42.00 ± 3.00	0.66 ± 0.03	64
KHP-1095	>100	0.05 ± 0.01	12.00 ± 1.00	0.05 ± 0.01	240
JDH-2110	>100	0.03 ± 0.01	9.30 ± 0.50	0.02 ± 0.01	465

PSI, phase specificity index.

Average values with SD are shown for at least three independent experiments. The PSI measures the ratio between experimental IC_50_ obtained at early and late phases of viral replication.

**Figure 6:**
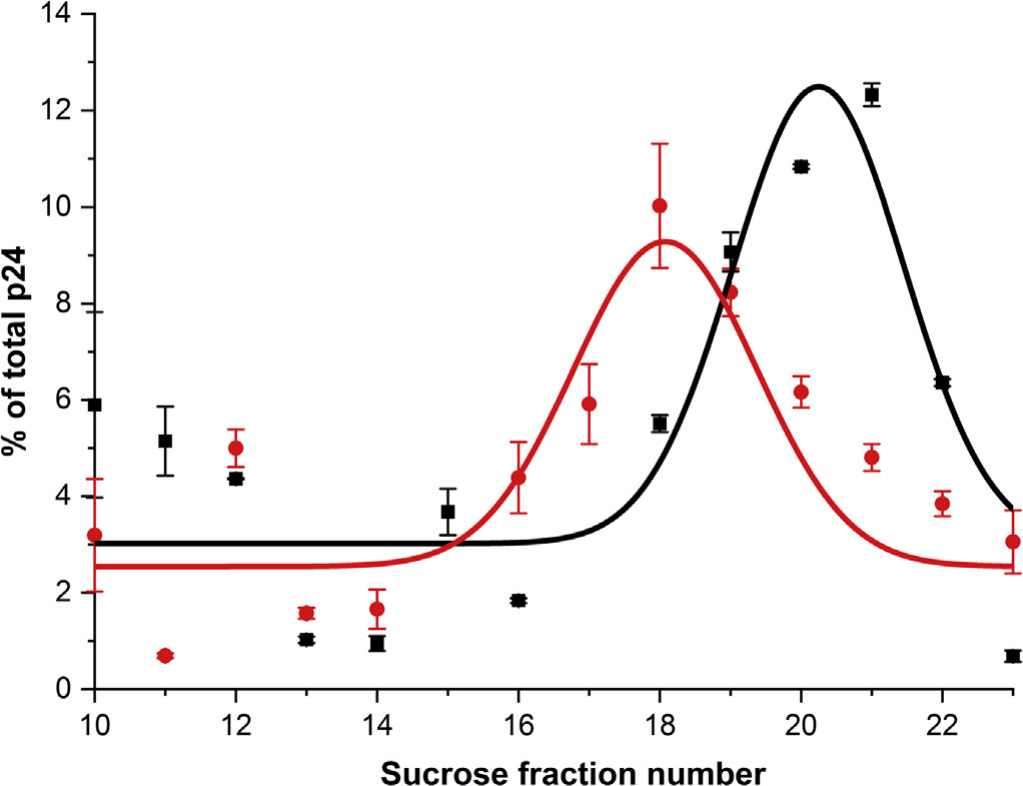
**Sucrose density gradient fractionation of detergent-lysed HIV-1 virions produced in the presence of DMSO (*black*) or 1 μM JDH2110 (*red*).** The graph represents the relative distribution of HIV-1 capsid (p24) in each of the sucrose density gradient fractions (average values of triplicate experiments and corresponding SDs are shown). HIV-1, human immunodeficiency virus type 1.

We and others have previously observed how ALLINIs interfere with proper virus particle maturation and yield noninfectious particles with eccentrically positioned RNPs. This mislocalization of the RNP to a position located between the low density empty capsid (CA) core and the particle membrane in mature virions has been observed using electron microscopy (17, 31, 32). The buoyant sucrose gradient technique that measure the relative density of the viral core of mature virions has also been validated as an effective approach to characterize this mislocalization of the RNP (17, 33, 34). Here, to confirm the effect of JDH2110 on virus particle maturation, we subjected cell-free and detergent-lysed progeny virions to a linear 20 to 70% (wt/vol) sucrose gradient fractionation. The CA core density was then detected by measuring the HIV-1 CA (p24) content of each sucrose fraction. This experiment revealed that the JDH2110 treatment during viral production resulted in reduction of CA signal in high-density fractions (compare fractions 16–22 in the absence and presence of JDH2110 in Fig. 6). These results show that the density of the viral cores strongly decreased upon JDH2110 treatment and are consistent with the formation of an empty core because of mislocalization of the RNPs (detected at fractions 15 in the presence of JDH2110 in Fig. 6).

## Discussion

The ALLINI class is composed of small molecules with a dual mode of action resulting from their binding location at the site where the cellular cofactor LEDGF/p75 interacts with IN. Previous structural studies have shown that ALLINIs are anchored to one side of the IN dimer interface through hydrogen bonds with IN residues H171 and T174. The other side of the IN dimer is engaged by a series of hydrophobic interactions between the quinoline scaffold and residues A128/A129. Additional contacts between an aryl group on position 4 and residues W132/L102 further anchor the compound into the V-shaped pocket and enhance inhibitory properties (Fig. 2). While originally designed to block IN-LEDGF/p75 interactions, these compounds also impact IN oligomeric structure and enzymatic activities through an IN multimerization mechanism (15).

By using computer simulations (25), crystallography (26), and biochemical experiments (27, 28), it has been suggested that in addition of binding at the IN CCD dimer interface, some ALLINIs would also contact the CTD of a third IN subunit, a feature that could be important for the development of the antiviral potency of this class of inhibitors. Interestingly, the ALLINIs’ overall activities on viral replication result from both early inhibition at the vDNA integration step and late effects during the maturation of the viral particle. *In vitro*, these compounds have been shown to display dual biochemical properties as they inhibit both the formation of the IN-LEDGF/p75 complex and induce aberrant IN multimerization. Several studies have revealed that the knockout of cellular LEDGF/p75 increased the potency of ALLINIs during the early stage of replication but had minimal effect when inhibitor exposure was limited to the late stage (32, 35, 36, 37). These results indicate that during integration, LEDGF/p75 competes with ALLINIs for the binding to IN and hence reduce inhibitor potency. However, this competition is notably absent or minimal during the late stage of HIV-1 replication, which allows ALLINIs to unrestrictedly induce IN multimerization.

Because aberrant IN multimerization within the maturing virions compromises the ability of IN to bind the viral RNA genome, these compounds induce mislocalization of RNPs outside of the conical core made of CA proteins and produces noninfectious virions (33). Because ALLINIs bind to the LEDGF-binding pocket at the IN dimer interface, it raises the question whether the two distinct biochemical and antiviral activities of these compounds are irreversibly linked or can be decoupled. In this study, we show that the nature of substitution at position 7 of the quinoline-based ALLINI can be optimized for specific biochemical and antiviral properties through an increase of interaction with a third IN subunit. Previous studies had shown the *in vitro* IN multimerization assay to be a reliable predictive tool to predict the antiviral activities of this class of compound (15, 17, 30, 31). Thus, using this HTRF-based assay, we tested several derivatizations of the scaffold at position 7, aiming to increase compound-induced IN multimerization through additional interactions with the residues Y226 and W235 of the CTD of a third subunit. We found that indeed the nature of the substitution at this position significantly impacts the *in vitro* IN multimerization EC_50_. A decrease in multimerization EC_50_ value was observed by adding a benzyl group at that location which was further enhanced by the coupling of either ortho- or para-substituted phenyl groups (Table 2).

Overall antiviral activity measurements with the ortho-methoxybenzene derivative (JDH2110) resulted in improved IC_50_ values about 30-fold compared with the unsubstituted quinoline scaffold (NGJ9002) (Table 3). Because ALLINIs can affect both integration and maturation steps, we obtained IC_50_ values at either early and late stages of viral replication by adding the inhibitor to target or producer cells. As previously observed for some (but not all) ALLINIs, we found that the compounds of our quinoline series were more effective during the late stage (Fig. 5*B*). Analytical sucrose density gradient fractionation of detergent-lysed virions and detection with p24 antibodies revealed that the JDH2110 treatment resulted in a significant shift of the viral capsid core density toward lower levels. These results indicate that the density of the viral cores decreased upon inhibitor treatment and are consistent with the formation of an empty core because of mislocalization of the RNPs (Fig. 6).

To quantify the amplitude between early and late stages, we defined a PSI that measures the ratio between experimental IC_50_s obtained at early and late phases of viral replication. We found that adding an ortho-substituted methoxy-benzene group at position 7 of the quinoline scaffold not only improve antiviral properties but also increased the PSI by 7-fold (Table 3, NGJ9002 *versus* JDH2110). To understand the specific molecular features important for the activity of our substituted quinoline series, we have performed several computer simulation experiments using the only available crystal structure (pdb: 5HOT) of a ALLINI-induced IN tetrameric assembly (26) as starting point. In the obtained calculated models, the addition of a substituted benzyl group at position 7 of the quinoline scaffold was shown to maximize hydrophobic interactions with aromatic residues Y226 and W235 of the third subunit (magenta in Fig. 4*A*). Furthermore, calculated binding energy extracted from the models indicated strong correlations with the experimentally measured multimerization EC_50_ values (Fig. 4*B*).

In summary, we have shown that targeted derivatization of our quinoline scaffold at position 7 significantly enhance the antiviral properties of these compound with a selectivity toward viral maturation and put this series in par with the potencies previously observed with pyridine-based ALLINIs (17). Because previous studies in cell culture have shown that combinations of ALLINIs and the FDA-approved active site IN inhibitor raltegravir displayed synergetic effects to inhibit HIV-1 (23), there are potential clinical benefits of combining these two classes of IN inhibitors to treat HIV-1 infected patients.

## Experimental procedures

### Synthesis of ALLINIs

The NGJ9002 compound was synthesized as described previously (19, 38). The synthesis of the multisubstituted quinolines series is detailed in the Supporting information.

### Recombinant proteins and IN multimerization assay

For biochemical studies, recombinant proteins 6xHis-tagged and FLAG-tagged full-length INs were expressed in *E. coli* and purified by column chromatography as previously described (39). The HTRF-based assay used to monitor inhibitor-induced aberrant multimerization of IN was performed as previously reported (15, 19). Briefly, two separate preparations of His-tagged and FLAG-tagged IN proteins were mixed in presence of increasing concentration of the test compounds and incubated for 2.5 h at room temperature. Anti-His6-XL665 and anti-FLAG-EuCryptate antibodies (Cisbio, Inc) were then added to the reaction and incubated at room temperature for 3 h. The IN multimerization HTRF signal was recorded using a PerkinElmer EnSpire multimode plate reader, and dose–response curves were fitted with a sigmoidal dose–response equation with Hill slope to determine the compound EC_50_ using Origin software (v9.7).

### Recombinant CTD-IN and binding assay with full-length IN

The CTD-IN protein was generated and purified using a published method (40). To measure the interaction of CTD-IN with full-length IN, His-tagged CTD-IN and FLAG-tagged full-length IN proteins were mixed in presence of DMSO or test compounds diluted in DMSO and incubated for 2.5 h at room temperature. Anti-His6-XL665 and anti-FLAG-EuCryptate antibodies (Cisbio, Inc) were then added to the reaction and incubated at room temperature for 3 h. The HTRF binding signal between proteins was recorded using a PerkinElmer EnSpire multimode plate reader.

### Antiviral and cytotoxicity assays

The EC_50_ values were determined in single replication cycle as described previously (17). Briefly, HEK293T cells were transfected with pNL4-3.Luc.Env- and pHEF-VSV-G plasmids to produce respective viruses in the absence or presence of indicated concentrations of ALLINIs. The virus supernatants were collected 24 h after drug treatment, and p24 concentrations were determined by HIV-1 Gag p24 ELISA (ZeptoMetrix) following manufacturer’s protocol. The target cells were then infected with HIV-1 virions equivalent to 4 ng of HIV-1 p24, in the absence or presence of indicated concentrations of drugs. To measure EC_50_ values during the early replication stage, DMSO or the inhibitor was added to the target cells 60 min before infection. The cells were cultured for 48 h, and the cell extracts were prepared using reporter lysis buffer (Promega). Luciferase activity was determined using a commercially available kit (Promega). The cytotoxicity assays were performed on noninfected cells using the commercially available CyQUANT XTT Cell Viability Assay (Invitrogen, Inc) by following the manufacturer protocol. The dose–response curves were fitted with a sigmoidal dose–response equation with Hill slope to determine the compound IC_50_ and CC_50_ using Origin software (v9.7).

### HIV-1 viral core analyses using buoyant sucrose density gradient fractionation

For sucrose density gradient fractionation (17, 33, 34), cell-free virions produced from HEK293T cells transfected with pNL4-3 in the absence or presence of indicated concentration of ALLINI were concentrated by ultracentrifugation over a 25% sucrose cushion, pelleted virions were lysed with 0.5% Triton X-100 and were ultracentrifuged through a 20% to 70% (wt/vol) linear sucrose density gradient at 28,500 rpm for 16 h at 4C in SW40 rotor (Beckman). Twenty-three 0.5 ml fractions were collected starting from the top of the gradient. The fractions were subjected to SDS-PAGE and Western blot using a p24 antibody. The relative distribution of HIV-1 capsid was determined by quantifying the Western blot p24 signal. The distributions were fitted with a Gauss equation using Origin software (v9.7).

### Molecular modeling

Molecular modeling was carried out using the Biovia Discovery Studio 2019 software package (Dassault Systèmes Biovia Inc) and the recent 4.4 Å X-ray structure 5HOT (26). The original isoquinoline ligand was replaced with NGJ9002. Symmetry operation was used to ensure the correct placement of CTD concerning the ligand and to resolve any changes in CTD position that occurred with ligand modification. NGJ9002 was allowed to be flexible and harmonic restraint was applied to the HIV-1 IN residues within 4.5 Å of NGJ9002. Any remaining residues surrounding 9.5 Å NGJ9002 were fixed. Additionally, the following distance restraints were used to ensure correct placement of ligand during standard dynamic cascade and to ensure that ligand remains within the binding pocket: the carboxylic acid group on C3 position and the E170 backbone of CCD, the carboxylic acid group on C3 position and the A169 backbone of CCD, the carboxylic acid group on C3 position and the K266 side-chain of CTD, and the methyl substituent on the C2 position and H171 side-chain. Using a methodology previously described (41, 42, 43), all distant residues outside of 9.5 Å shell were excluded from the calculation during standard dynamic cascade to optimize simulation speed. Standard dynamic cascade runs were performed with 1000 iteration of Steepest Descent minimization followed by 2000 iterations of conjugated gradient minimalization at 0.1 RMS gradient. Simulated heating runs were performed, from 800 to 298 K with a simulation time of 5 ps at 0.5 fs/step. This was followed by equilibration with 10 ps simulation time at 0.5 fs/step. Finally, the production run was carried out with 20 ps simulation time at 0.5 fs/step. A 9.5-Å atom-based cut-off for nonbonding interactions was used during the standard dynamic cascade calculations with the dielectric constant set at 2.0. After the final round of molecular dynamics, the lowest energy conformation structure was selected. The ligand was isolated and manually defined for the binding energy calculations. The structure was minimized to a final convergence criterion of 0.001, using Smart minimization consisting of 1000 iterations of the Steepest Descent method followed by 2000 iterations of the conjugate gradient in succession using the integrated binding energy calculation program. The above steps were repeated for each of the final compounds in the series.

## Data availability

All data are contained within the manuscript.

## Conflict of interests

The authors declare that they have no conflicts of interest with the contents of this article.
