# Supplementary material for: Optimized binding of substituted quinoline ALLINIs within the HIV-1 integrase oligomer
Source: J Biol Chem. 2021 Feb 2;296:100363. doi: 10.1016/j.jbc.2021.100363 (PMC7949159; doi:10.1016/j.jbc.2021.100363)
Supplement: Supporting information [file mmc1.pdf]

SUPPORTING INFORMATION

Optimized binding of substituted quinoline ALLINIs within the HIV-1 integrase oligomer.

Jian Sun, Krunal Patel, Jared Hume, Julie A. Pigza, Matthew G. Donahue and Jacques J. Kessl

Unless otherwise noted all reactions were run under a positive atmosphere of nitrogen in oven-dried or flame-dried conical or round bottom microwave vials capped with PTFE-lined caps. Solvents were removed by rotary evaporation at temperatures lower than 45 °C. ^{1}H NMR spectra were collected on 400 MHz Bruker instrument using $CDCl_3$ which was referenced at 7.26 ppm (residual chloroform proton), and CD_3OD referenced at 3.31 ppm (residual methanol proton) as the solvents. ^{13}C NMR spectra were collected using same NMR instrument using $CDCl_3$ referenced at 77 ppm and CD_3OD referenced at 49 ppm. Unless specified, purification was performed using an ISCO Prep HPLC with C18 column (0-100% acetonitrile in H2O gradient). All compounds were isolated as racemic mixtures containing two diastereomers. For all following compounds, only the ^{1}H NMR peaks of the major diastereomer are listed. Deuterium exchange of the carboxylic acid hydrogen in CD_3OD was exchanged for the S4 compounds.

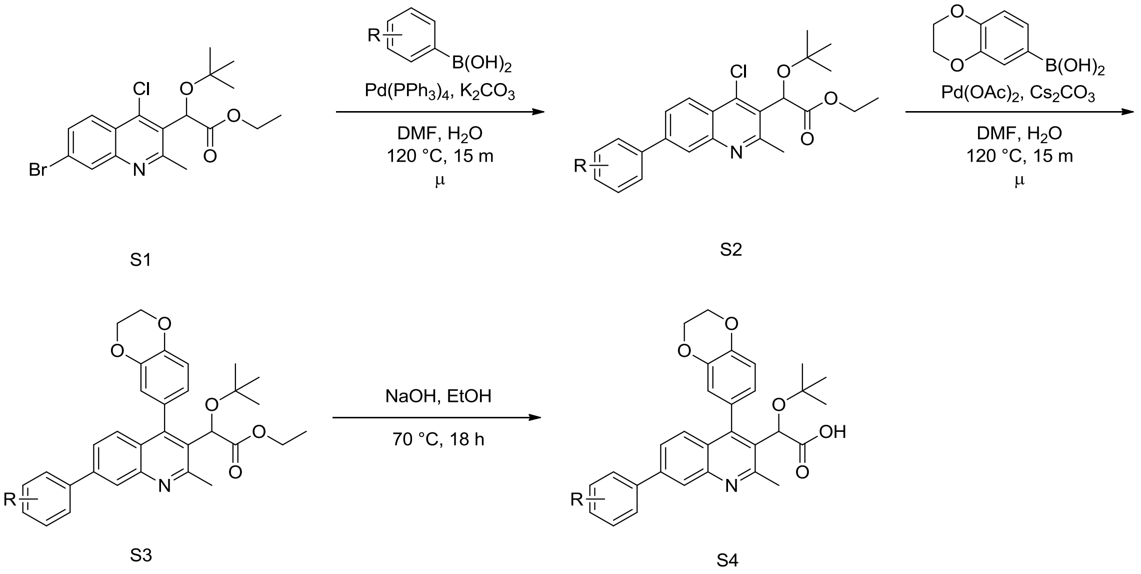

Scheme 1. Synthetic route for the preparation of 7-position aryl quinoline scaffold derivatives.

Entry	R
a	H
b	2-Cl
c	2-CH_3
d	2-CF_3
e	2-OCH_3
f	3-OCH_3
g	4-OCH_3
h	2-OCF_3
i	2-OCH_2CH_3

General Methods.
General procedure A: Preparation of quinolines with aryl derivatives on the 7-position **S2**. Suzuki cross coupling reactions were carried out by adding **S1** (1 equiv), a substituted phenyl boronic acid (1.1 equiv), $Pd(PPh_3)_4$ (0.2 equiv), and K_2CO_3 (3.0 equiv) in that order to a solution of H_2O (3.3 M) and DMF (0.33 M) in a 0.5 mL – 2.0 mL size microwave vial with stir bar. The vial was capped, and the mixture was stirred vigorously for 5 min. The microwave vial containing the reaction mixture was placed in the Biotage® Initiator+ microwave synthesizer at 120 °C for 15 minutes. After cooling the reacted mixture to room temperature, the mixture was filtered through Celite and washed with approximately 5 mL EtOAc. The filtrate was transferred to a separatory funnel and extracted with EtOAc (3 x 2 mL). The combined organic phases were washed with H_2O and then brine. The organic phase was dried over Na_2SO_4 and concentrated under reduced pressure. The final compound was purified using column chromatography as noted in each experimental.

General procedure B: Preparation of quinolines with a benzodioxane derivative at the 4-position **S3**. Suzuki cross coupling reactions were carried out by adding **S2** (1 equiv), 1,4-benzodioxane-6-boronic acid (1.1 equiv), Cs_2CO_3 (3.0 equiv), and $Pd(OAc)_2$ (0.2 equiv) in that order to a solution of H_2O (3.3 M) and DMF (0.33 M) in a 0.5 mL – 2.0 mL size microwave vial with stir bar. The vial was capped and the reaction mixture was stirred vigorously for 5 min. Unless stated otherwise, the microwave vial containing the reaction mixture was placed in the Biotage® Initiator+ microwave synthesizer at 120 °C for 15 minutes. After cooling the reacted mixture to room temperature, the mixture was filtered through Celite and washed with approximately 5 mL EtOAc. The filtrate was transferred to a separatory funnel and extracted with EtOAc (3 x 2 mL). The combined organic phases were washed with H_2O and then brine. The organic phase was dried over Na_2SO_4 and concentrated under reduced pressure. The final compound was purified using column chromatography as noted in each experimental.

General procedure C: Saponification reactions to form **S4**. To a solution of 10.0 M NaOH (10.0 equiv) and EtOH (0.25 M) in a 0.5 mL – 2.0 mL size microwave vial with stir bar was added **S3** (1 equiv) and the vial was capped. The reaction mixture was heated to 70 °C in a fitted metal heating block and stirred overnight until the solution changed color to dark brown. After cooling to room temperature, the reaction was quenched with 1N HCl solution to an approximate pH of 4.0. The newly formed precipitate was filtered using buchner funnel and filtrate was discarded. Crude material was rinsed with approximately 5 mL of MeOH to remove any remaining salts formed during quenching. The final compound was purified using preparative HPLC as noted above.

Preparation of **Ethyl 2-(tert-butoxy)-2-(4-chloro-2-methyl-7-phenylquinolin-3-yl) acetate S2a**. Applying general procedure A to 2-(7-bromo-4-chloro-2-methylquinolin-3-yl)-2-(tert-butoxy) acetate (**S1**) (26.0 mg, 62.9 µmoles), phenyl boronic acid (9.9 mg, 81.2 µmoles), K_2CO_3 (25.0 mg, 180.9 µmoles), and $Pd(PPh_3)_4$ (14.9 mg, 12.9 µmoles) in a solution of DMF (731 µL) and H_2O (73.1µL) afforded, after flash chromatography (SiO_2, 0-100% ethyl acetate in hexanes gradient), **S2a** (16.8 mg, 78%) as an orange oil: ^1H NMR (400 MHz, $CDCl_3$) δ 8.29 (d, *J* = 8.7 Hz, 1H), 8.25 (d, *J* = 1.3 Hz, 1H), 7.87 (dd, *J* = 8.7, 1.7 Hz, 1H), 7.76 (dd, *J* = 7.5, 1.3 Hz, 2H), 7.51 (t, *J* = 7.5

Hz, 2H), 7.42 (tt, J = 7.4, 1.2 Hz, 1H), 5.99 (s, 1H), 4.20 (dq, J = 7.1, 1.0 Hz, 1H), 4.19 (dq, J = 7.1, 1.0 Hz, 1H), 2.82 (s, 3H), 1.26 (s, 9H), 1.19 (t, J = 7.1 Hz, 3H). The diastereomeric ratio (dr) could not be determined.

Preparation of **Ethyl 2-(tert-butoxy)-2-(4-(2,3-dihydrobenzo[b][1,4] dioxin-6-yl)-2-methyl-7-phenylquinolin-3-yl) acetate S3a**. Applying general procedure B to **S2a** (16.8 mg, 40.9 µmoles), 1,4-benzodioxane-6-boronic acid (8.1 mg, 45.0 µmoles), K_2CO_3 (17.5 mg, 126.6 µmoles), and $Pd(OAc)_2$ (2.1 mg, 9.4 µmoles) in a solution of DMF (731 µL) and H_2O (73.1µL) afforded, after flash chromatography (SiO_2, 0-100% ethyl acetate in hexanes gradient), **S3a** (58%) as an orange oil: ^{1}H NMR (400 MHz, $CDCl_3$) δ 8.27 (d, J = 1.7 Hz, 1H), 7.74 (dd, J = 7.2, 1.3 Hz, 2H), 7.63 (dd, J = 8.7, 1.9 Hz, 1H), 7.55 - 7.46 (m, 3H), 7.42 - 7.36 (m, 1H), 7.06 - 6.96 (m, 2H), 6.89 - 6.80 (m, 1H), 5.26 (s, 1H), 4.41 - 4.35 (m, 4H), 4.27 - 4.14 (m, 2H), 2.85 (s, 3H), 1.23 (t, J = 6.7 Hz, 3H), 1.02 (s, 9H). The diastereomeric ratio (dr) of 1.3:1 was determined based on the peaks at 1.02 and 1.01 ppm.

Preparation of **2-(tert-butoxy)-2-(4-(2,3-dihydrobenzo[b][1,4] dioxin-6-yl)-2-methyl-7-phenylquinolin-3-yl) acetic acid S4a**. Applying general procedure C to **S3a** (54.0 mg, 105.6 µmoles) and ethanol (123.4 µL) in a solution of 10.0 M NaOH (105.6 µL) afforded, after prep HPLC on Xbridge™ Prep Shield Rp18, 10 µM OBD™ 19 X 250mm column (eluted with 0-100% HPLC grade submicron filtered water and Acetonitrile gradient), **S4a (**31.8 mg, 62%) as a white powder: ^{1}H NMR (400 MHz, MeOD) δ 8.20 (s, 1H), 7.81 - 7.77 (m, 3H), 7.60 (d, J = 8.8 Hz, 1H), 7.50 (t, J = 7.7 Hz, 2H), 7.41 (t, J = 7.3 Hz, 1H), 7.18 - 7.13 (m, 1H), 7.09 - 7.04 (m, 1H), 6.86 - 6.84 (m, 1H), 5.34 (s, 1H), 4.43 - 4.29 (m, 4H), 2.84 (s, 3H), 1.00 (s, 9H). The diastereomeric ratio (dr) of 1.3:1 was determined based on the peaks at 1.01 and 1.00 ppm.

Preparation of **Ethyl 2-(tert-butoxy)-2-(4-chloro-7-(2-chlorophenyl)-2-methylquinolin-3-yl) acetate S2b**. Applying general procedure A to Ethyl 2-(7-bromo-4-chloro-2-methylquinolin-3-yl)-2-(tert-butoxy) acetate (**S1**) (100.5 mg, 241.13 µmoles), 2-chlorophenyl boronic acid (46.6 mg, 289.35 µmoles), K_2CO_3 (105.6 mg, 723.38 µmoles), $Pd(PPh_3)_4$ (56.5 mg, 48.23 µmoles) in a solution of DMF (731 µL) and H_2O (73.1µL) afforded, after flash chromatography (SiO_2, 0-100% ethyl acetate in hexanes gradient), **S2b**(30.5 mg, 47%) as an orange oil: ^{1}H NMR (400 MHz, $CDCl_3$) δ 8.29 (d, J = 8.7 Hz, 1H), 8.25 (d, J = 1.7 Hz, 1H), 7.87 (dd, J = 1.8, 8.7 Hz, 1H), 7.78 - 7.74 (m, 1H), 7.51 (t, J = 7.5 Hz, 2H), 7.42 (t, J = 7.3 Hz, 1H), 5.99 (s, 1H), 4.18 (q, J = 7.1 Hz, 2H), 2.82 (s, 3H), 1.26 (s, 9H), 1.19 (t, J = 7.1 Hz, 3H). The diastereomeric ratio (dr) could not be determined.

Preparation of **Ethyl 2-(tert-butoxy)-2-(7-(2-chlorophenyl)-4-(2,3-dihydrobenzo[b][1,4] dioxin-6-yl)-2-methylquinolin-3-yl)acetate S3b**. Applying general procedure B to **S2b** (30.1 mg, 68.33 µmoles), 1,4-benzodioxane -6- boronic acid (14.0 mg, 75.16 µmoles), K_2CO_3 (32.1 mg,

204.99 µmoles), Pd(OAc)$_2$ (3.7 mg, 13.67 µmoles) in a solution of DMF (731 µL) and H_2O (73.1µL) afforded, after flash chromatography (SiO_2, 0-100% ethyl acetate in hexanes gradient), **S3b** (14.4 mg, 39%) as an orange oil: ^{1}H NMR (400 MHz, $CDCl_3$) δ 8.09 (d, *J* = 0.8 Hz, 1H), 7.54 - 7.51 (m, 1H), 7.51 - 7.47 (m, 2H), 7.47 - 7.41 (m, 1H), 7.38 - 7.29 (m, 2H), 7.06- 6.97 (m, 2H), 6.91 - 6.82 (m, 1H), 5.26 (s, 1H), 4.46 - 4.30 (m, 4H), 4.30 - 4.10 (m, 2H), 2.84 (s, 3H), 2.83 (s, 3H), 1.25 (t, *J* = 6.8 Hz, 3H), 1.02 (s, 9H). The diastereomeric ratio (dr) of 1.2:1 was determined based on the peaks at 1.02 and 1.03 ppm.

Preparation of **2-(tert-butoxy)-2-(7-(2-chlorophenyl)-4-(2,3-dihydrobenzo[b][1,4] dioxin-6-yl)-2-methylquinolin-3-yl) acetic acid S4b**. Applying general procedure C to **S3b** (14.4 mg, 46.07 µmoles) and ethanol (1.0 mL) in a solution of 10.0 M NaOH (500.0 µL) afforded, after prep HPLC on XbridgeTM Prep Shield Rp18, 10 µM OBDTM 19 X 250mm column (eluted with 0-100% HPLC grade submicron filtered water and Acetonitrile gradient), **S4b (**6.2 mg, 43%) as white powder: ^{1}H NMR (400 MHz, MeOD) δ 8.18 (s, 1H), 7.88 - 7.75 (m, 2H), 7.60 (d, *J* = 4.2 Hz, 1H), 7.56 - 7.52 (m, 1H), 7.51 - 7.45 (m, 2H), 7.19 - 7.10 (m, 2H), 7.03 - 6.93 (m, 1H), 5.43 (s, 1H), 4.42 - 4.32 (m, 4H), 3.05 (s, 3H), 1.04 (s, 9H). The diastereomeric ratio (dr) of 1.4:1 was determined based on the peaks at 1.05 and 1.04 ppm.

Preparation of **Ethyl 2-(tert-butoxy)-2-(4-chloro-2-methyl-7-(o-tolyl)quinolin-3-yl) acetate S2c**. Applying general procedure A to ethyl 2-(7-bromo-4-chloro-2-methylquinolin-3-yl)-2-(tert-butoxy) acetate (**S1**) (101.4 mg, 241.13 µmoles), 2-methylphenyl boronic acid (39.5 mg, 289.35 µmoles), K_2CO_3 (100.1 mg, 723.38 µmoles), Pd(PPh$_3$)$_4$ (55.9 mg, 48.23 µmoles) in a solution of DMF (731 µL) and H_2O (73.1µL) afforded, after flash chromatography (SiO_2, 0-100% ethyl acetate in hexanes gradient), **S2c** (76.7 mg, 79%) as an orange oil: ^{1}H NMR (400 MHz, $CDCl_3$) δ 8.26 (d, *J* = 8.6 Hz, 1H), 7.97 (d, *J* = 1.4 Hz, 1H), 7.59 (dd, *J* = 1.7, 8.6 Hz, 1H), 7.33 - 7.29 (m, 4H), 6.00 (s, 1H), 4.20 (dq, *J* = 7.1, 1.0 Hz, 1H), 4.19 (dq, *J* = 7.1, 1.0 Hz, 1H), 2.82 (s, 3H), 2.33 (s, 3H), 1.26 (s, 9H), 1.19 (t, *J* = 7.1 Hz, 3H). The diastereomeric ratio (dr) could not be determined.

Preparation of **Ethyl 2-(tert-butoxy)-2-(4-(2,3-dihydrobenzo[b][1,4]dioxin-6-yl)-2-methyl-7-(o-tolyl)quinolin-3-yl) acetate S3c**. Applying general procedure B ot **S2c** (76.6 mg, 179.83 µmoles), 1,4-benzodioxane -6- boronic acid (8.1 mg, 45.0 µmoles), K_2CO_3 (77.3 mg, 539.50 µmoles), Pd(OAc)$_2$ (8.1 mg, 35.97 µmoles) in a solution of DMF (731 µL) and H_2O (73.1µL) afforded, after flash chromatography (SiO2, 0-100% ethyl acetate in hexanes gradient), **S3c** (32.4 58%) as an orange oil: ^{1}H NMR (400 MHz, $CDCl_3$) δ 7.99 (d, *J* = 1.6 Hz, 1H), 7.47 (d, *J* = 16.0 Hz, 1H), 7.35 (dd, *J* = 1.7, 8.6 Hz, 1H), 7.33 - 7.27 (m, 4H), 7.07 - 6.98 (m, 2H), 6.91 - 6.83 (m, 1H), 5.26 (s, 1H), 4.42 - 4.31 (m, 4H), 4.29 - 4.12 (m, 2H), 2.84 (s, 3H), 2.33 (s, 3H), 1.25 (t, *J* = 6.8 Hz, 3H), 1.02 (s, 9H). The diastereomeric ratio (dr) of 1.2:1 was determined based on the peaks at 1.03 and 1.02 ppm.

Preparation of **2-(tert-butoxy)-2-(4-(2,3-dihydrobenzo[b][1,4]dioxin-6-yl)-2-methyl-7-(o-tolyl)quinolin-3-yl) acetic acid S4c**. Applying general procedure C to to **S3c** (32.4 mg, 61.64 µmoles) and ethanol (1.0 mL) in a solution of 10.0 M NaOH (500.0 µL) afforded, after prep HPLC on Xbridge^{TM} Prep Shield Rp18, 10 µM OBD^{TM} 19 X 250mm column (eluted with 0-100% HPLC

grade submicron filtered water and Acetonitrile gradient), **S4c (**31.8 mg, 47%) as pale yellow powder: ^{1}H NMR (400 MHz, MeOD) δ 7.90 (d, J = 1.4 Hz, 1H), 7.54 (d, J = 8.6 Hz, 1H), 7.46 (dd, J = 8.6, 1.7 Hz, 1H), 7.34 - 7.24 (m, 4H), 7.20 - 7.11 (m, 1H), 7.10 - 7.01 (m, 1H), 6.93 - 6.83 (m, 1H), 5.36 (s, 1H), 4.44 - 4.29 (m, 4H), 2.84 (s, 3H), 2.29 (s, 3H), 1.01 (s, 9H). The diastereomeric ratio (dr) of 1.2:1 was determined based on the peaks at 1.02 and 1.01 ppm.

Preparation of **Ethyl 2-(tert-butoxy)-2-(4-chloro-2-methyl-7-(2-(trifluoromethyl) phenyl) quinolin-3-yl) acetate S2d**. Applying general procedure A to Ethyl 2-(7-bromo-4-chloro-2-methylquinolin-3-yl)-2-(tert-butoxy) acetate (**S1**) (102.9 mg, 241.13 µmoles), 2-trifloromethyl phenyl boronic acid (50.5 mg, 265.24 µmoles), K_2CO_3 (100.4.0 mg, 723.38 µmoles), $Pd(PPh_3)_4$ (21.0 mg, 48.23 µmoles) in a solution of DMF (731 µL) and H_2O (73.1µL) afforded, after flash chromatography (SiO_2, 0-100% ethyl acetate in hexanes gradient), **S2d** (75.5 mg, 67%) as an orange oil: ^{1}H NMR (400 MHz, $CDCl_3$) δ 8.26 (d, *J* = 8.7 Hz, 1H), 7.99 (d, *J* = 1.5 Hz, 1H), 7.80 (d, *J* = 7.7 Hz, 1H), 7.64 - 7.49 (m, 3H), 7.44 - 7.37 (m, 1H), 6.01 (s, 1H), 4.20 (dq, *J* = 7.1, 1.9 Hz, 1H), 4.19 (dq, *J* = 7.1, 1.9 Hz, 1H), 2.82 (s, 3H), 1.27 (s, 9H), 1.20 (t, *J* = 7.1 Hz, 3H). The diastereomeric ratio (dr) could not be determined.

Preparation of **Ethyl 2-(tert-butoxy)-2-(4-(2,3-dihydrobenzo[b][1,4] dioxin-6-yl)-2-methyl-7-(2-(trifluoromethyl)phenyl)quinolin-3-yl)acetate S3d**. Applying general procedure B to **S2d** (89.70 mg, 180.88 µmoles) 1,4-benzodioxane -6- boronic acid (35.81 mg, 198.96 µmoles), K_2CO_3 (74.99 mg, 542.63 µ,moles), $Pd(OAc)_2$ (8.1 mg, 36.18 µmoles) in a solution of DMF (731 µL) and H_2O (73.1µL) afforded, after flash chromatography (SiO_2, 0-100% ethyl acetate in hexanes gradient), **S2d** (21.9 mg, 21%) as an orange oil: ^{1}H NMR (400 MHz, $CDCl_3$) δ 7.99 (d, *J* = 1.3 Hz, 1H), 7.79 (d, *J* = 7.9 Hz, 1H), 7.59 (t, *J* = 7.5 Hz, 1H), 7.53 - 7.33 (m, 4H), 7.06 - 7.00 (m, 2H), 6.91 - 6.83 (m, 1H), 5.27 (s, 1H), 4.39 - 4.35 (m, 4H), 4.26 - 4.14 (m, 2H), 2.84 (s, 3H), 1.27 (t, *J* = 7.1 Hz, 3H), 1.03 (s, 9H). The diastereomeric ratio (dr) of 1.2:1 was determined based on the peaks at 1.03 and 1.02 ppm.

Preparation of **2-(tert-butoxy)-2-(4-(2,3-dihydrobenzo[b][1,4] dioxin-6-yl)-2-methyl-7-(2-(trifluoromethyl)phenyl)quinolin-3-yl)acetic acid S4d**. Applying general procedure C to **S3d** (21.9 mg, 42.81 µmoles) and ethanol (44.12 µL) in a solution of 10.0 M NaOH (37.78 µL) afforded, after prep HPLC on Xbridge™ Prep Shield Rp18, 10 µM OBD™ 19 X 250mm column (eluted with 0-100% HPLC grade submicron filtered water and Acetonitrile gradient), **S4d** (7.8 mg, 37%) as white solid powder: ^{1}H NMR (400 MHz, MeOD) δ 7.82 (s, 1H), 7.73 (d, *J* = 7.8 Hz, 1H), 7.59 (t, *J* = 7.6 Hz, 1H), 7.51 (t, *J* = 7.5 Hz, 1H), 7.47 - 7.40 (m, 1H), 7.39 - 7.29 (m, 2H), 7.06 - 6.93 (m, 2H), 6.79 - 6.75 (m, 1H), 5.26 (s, 1H), 4.30 - 4.20 (m, 4H), 2.73 (s, 3H), 0.91 (s, 9H). The diastereomeric ratio (dr) of 1.4:1 was determined based on the peaks at 0.92 and 0.91 ppm.

Preparation of **3-(1-(tert-butoxy)-2-ethoxyallyl)-4-chloro-7-(2-methoxyphenyl)-2-methylquinoline S2e**. Applying modified general procedure A to Ethyl 2-(7-bromo-4-chloro-2-methylquinolin-3-yl)-2-(tert-butoxy) acetate (**S1**) (100.0 mg, 241.1 µmoles), 2-methoxy phenyl boronic acid (43.9 mg, 289.4 µmoles), K_2CO_3 (100.0 mg, 723.4 µmoles), $Pd(PPh_3)_4$ (55.73 mg,

48.23 µmoles) in a solution of DMF (731 µL) and H_2O (73.1µL) and reacted at 90 °C for 12 hours on heat plate, afforded, after flash chromatography (SiO_2, 0-100% ethyl acetate in hexanes gradient) **S2e** (77.8 mg, 73%) as an orange oil: ^{1}H NMR (400 MHz, $CDCl_3$) 8.23 (d, *J* = 8.7 Hz, 1H), 8.17 (d, *J* = 1.5 Hz, 1H), 7.82 (dd, *J* = 8.7, 1.6 Hz, 1H), 7.44 (m, 1H), 7.38 (m, 1H), 7.08 (m, 1H), 7.03 (d, 8.2 Hz, 1H), 5.99 (s, 1H), 4.37 (dq, *J* = 7.1, 1.9 Hz, 1H), 4.38 (dq, *J* = 7.1, 1.9 Hz, 1H) 3.84 (s, 3H), 2.81 (s, 3H), 1.25 (s, 9H), 1.18 (t, *J* = 7.0 Hz, 3H). The diastereomeric ratio (dr) could not be determined.

Preparation of **3-(1-(tert-butoxy)-2-ethoxyallyl)-4-(2,3-dihydrobenzo[b][1,4] dioxin-6-yl)-7-(2-methoxyphenyl)-2-methylquinoline S3e.** Applying modified general procedure B to **S2e** (77.8 mg,176.0 µmoles),1,4-benzodioxane -6- boronic acid (38.0 mg, 211.3 µmoles), K_2CO_3 (72.9 mg, 528.1 µmoles), $Pd(OAc)_2$ (7.9 mg, 35.9 µmoles) in a solution of DMF (731 µL) and H_2O (73.1µL) and reacted at 90 °C for 12 hours on heat plate, afforded, after flash chromatography (SiO_2, 0-100% ethyl acetate in hexanes gradient), **S3e** (58.9 mg, 55%) as an orange oil: ^{1}H NMR (400 MHz, $CDCl_3$) 8.24-8.17 (m, 2H), 7.83-7.80 (m, 1H), 7.62-7.57 (dd, *J* = 5.1, 1.6 Hz, 1H), 7.49 - 7.32 (m, 6H), 6.90-6.82 (m, 1H), 5.26 (s, 1H), 4.36 - 4.33 (m, 4H), 4.21 - 4.16 (m, 2H), 3.80 (s, 3H) 2.84 (s, 3H), 2.81 (s, 3H), 1.17 (t, *J* = 2.7 Hz, 3H), 1.01 (s, 9H). The diastereomeric ratio (dr) of 1.3:1 was determined based on the peaks at 1.01 and 1.01 ppm.

Preparation of **3-(tert-butoxy)-3-(4-(2,3-dihydrobenzo[b][1,4] dioxin-6-yl)-7-(2-methoxyphenyl)-2-methylquinolin-3-yl) prop-1-en-2-ol S4e.** Applying general procedure C to **S3e** (58.9 mg, µmoles) and ethanol (1 mL) in a solution of 10.0 M NaOH (500 µL), afforded, after prep HPLC on Xbridge™ Prep Shield Rp18, 10 µM OBD™ 19 X 250mm column (eluted with 0-100% HPLC grade submicron filtered water and Acetonitrile gradient), **S4e** (4.2 mg, 8%) as a white solid powder: ^{1}H NMR (400 MHz, $CDCl_3$) 8.09 (d, *J*=2.5 Hz, 1H), 7.59 (t, *J* = 8.6 Hz, 1H), 7.55 - 7.49 (m, 2H), 7.45 - 7.40 (m, 1H), 7.39 - 7.36 (m, 1H), 7.15 - 7.13 (m, 1H), 7.08 (q, *J* = 5.0 Hz, 1H), 7.03 (d, *J* = 8.2 Hz, 1H), 6.88 - 6.84 (m, 1H), 5.22 (s, 1H), 4.39 - 4.30 (m, 4H), 3.84 (s, 3H), 2.89 (s, 3H), 0.98 (s, 9H). The diastereomeric ratio (dr) of 1.2:1 was determined based on the peaks at 0.987 and 0.985 ppm.

Preparation of **3-(1-(tert-butoxy)-2-ethoxyallyl)-4-chloro-7-(3-methoxyphenyl)-2-methylquinoline S2f.** Applying modified general procedure A to Ethyl 2-(7-bromo-4-chloro-2-methylquinolin-3-yl)-2-(tert-butoxy) acetate (**S1**) (100.0 mg, 241.1 µmoles), 3-methoxy phenyl boronic acid (43.9 mg, 289.4 µmoles), K_2CO_3 (100.0 mg, 723.4 µmoles), $Pd(PPh_3)_4$ (55.73 mg, 48.23 µmoles) in a solution of DMF (731 µL) and H_2O (73.1µL) reacted at 90 °C for 12 hours on heat plate, afforded, after flash chromatography (SiO_2, 0-100% ethyl acetate in hexanes gradient) **S2f** (72.6 mg, 68%) as an orange oil: ^{1}H NMR (400 MHz, $CDCl_3$) 8.28 (d, *J* = 8.8 Hz, 1H), 8.25 (d, *J* = 1.6 Hz, 1H), 7.86 (dd, *J* = 8.7, 1.8 Hz, 1H), 7.42 (m, 1H), 7.34 (m, 1H), 7.29 (m, 1H), 6.97 (m, 1H), 5.99 (s, 1H), 4.38 (dq, *J* =7.1, 1.0 Hz, 1H), 4.37 (dq, *J* = 7.1, 1.0 Hz, 1H)3.89 (s, 3H), 2.82 (s, 3H), 1.25 (s, 9H), 1.19 (t, *J* = 7.0 Hz, 3H). The diastereomeric ratio (dr) could not be determined.

Preparation of **3-(1-(tert-butoxy)-2-ethoxyallyl)-4-(2,3-dihydrobenzo[b][1,4] dioxin-6-yl)-7-(3-methoxyphenyl)-2-methylquinoline S3f.** Applying modified general procedure B mixture of **S2f** (72.6 mg, 164.2 µmoles), 1,4-benzodioxane-6-boronic acid (35.5 mg, 197.1 µmoles), K_2CO_3 (68.1 mg, 492.8 µmoles), $Pd(OAc)_2$ (7.3 mg, 32.8 µmoles) in a solution of DMF (731 µL) and H_2O (73.1µL) reacted at 90 °C for 12 hours on heat plate, afforded, after flash chromatography (SiO_2, 0-100% ethyl acetate in hexanes gradient) **S3f** (18.2 mg, 20%) as an orange oil: ^{1}H NMR (400 MHz, $CDCl_3$) δ 8.26 (d, *J* = 8.8 Hz, 1H), 8.21 (d, *J* = 1.0 Hz, 1H), 7.84 (dd, *J* = 8.7, 1.8 Hz, 1H), 7.70 (d, *J* = 8.8 Hz, 2H), 7.04 (d, *J* = 8.8 Hz, 2H), 5.98 (s,1H), 4.21 (dq, *J* = 7.1, 0.9 Hz, 1H), 4.19 (dq, *J* = 7.1, 0.9 Hz, 1H) 3.88 (s, 3H), 2.82 (s, 3H), 1.25 (s, 9H).The diastereomeric ratio (dr) could not be determined.

Preparation of **3-(tert-butoxy)-3-(4-(2,3-dihydrobenzo[b][1,4] dioxin-6-yl)-7-(3-methoxyphenyl)-2-methylquinolin-3-yl) prop-1-en-2-ol S4f.** Applying modified general procedure C to **S3f** (18.2mg, 39.9 µmoles) and ethanol (1 mL) in a solution of 10.0 M NaOH (500 µL) afforded, after prep HPLC on Xbridge™ Prep Shield Rp18, 10 µM OBD™ 19 X 250mm column (eluted with 0-100% HPLC grade submicron filtered water and Acetonitrile gradient), **S3f** (14.2 mg, 34%) as a white solid powder: ^{1}H NMR (400 MHz, $CDCl_3$) 8.21 (s, 1H), 7.76 (dd, *J* = 8.7, 1.4Hz, 1H), 7.57 - 7.49 (m, 2H), 7.41 (d, *J* = 8.0 Hz, 1H), 7.37 - 7.27 (m, 2H), 7.20 - 7.14 (m, 1H), 7.02 - 6.93 (m, 2H), 5.31 (s, 1H), 3.89 (s, 3H), 3.87 (s, 3H), 2.88 (s, 3H), 1.00 (s, 9H).The diastereomeric ratio (dr) of 1.4:1 was determined based on the peaks at 1.02 and 1.00 ppm.

Preparation of **Ethyl 2-(tert-butoxy)-2-(4-chloro-2-methyl-7-(2-(trifluoromethoxy) phenyl) quinolin-3-yl) acetate S2g**. Applying general procedure A to Ethyl 2-(7-bromo-4-chloro-2-methylquinolin-3-yl)-2-(tert-butoxy) acetate (**S1**) (100.0 mg, 241.12 µmoles), 4-methoxy phenyl boronic acid (62.29 mg, 409.91 µmoles), K_2CO_3 (101.2 mg, 186.0 µmoles), $Pd(PPh_3)_4$ (13.93 mg, 12.06 µmoles) in a solution of dioxane (2.0 mL) and H_2O (1.0 mL), afforded, after flash chromatography (SiO_2, 0-100% ethyl acetate in hexanes gradient), **S2g** (91.5 mg, 86%) as a yellow oil: ^{1}H NMR (400 MHz, $CDCl_3$) δ 8.28 (d, *J* = 8.7 Hz, 1H), 8.11 (d, *J* = 1.6 Hz, 1H), 7.74 (dd, *J* =1.7, 8.7 Hz, 1H), 7.55 (d, *J* = 2.1 Hz, 1H), 7.47 - 7.39 (m, 3H), 6.01 (s, 1H), 4.19 (dq, *J* = 7.1, 1.0 Hz, 1H), 4.18 (dq, *J* = 7.1, 1.0 Hz, 1H) 2.82 (s, 3H), 1.27 (s, 9H), 1.20 (t, *J* = 7.1 Hz, 3H). The diastereomeric ratio (dr) could not be determined.

Preparation of **Ethyl 2-(tert-butoxy)-2-(4-(2,3-dihydrobenzo[b][1,4] dioxin-6-yl)-2-methyl-7-(2-(trifluoromethoxy) phenyl) quinolin-3-yl) acetate S3g**. Applying general procedure B to **S2g** (91.50 mg, 207.04 µmoles), 1,4-benzodioxane-6-boronic acid (65.20 mg, 363.32 µmoles), Cs_2CO_3 (131.91 mg, 414.08 µmoles), $Pd(OAc)_2$ (2.32 mg, 10.35 µmoles) in a solution of dioxane (2.0 mL) and H_2O (1.0 mL) afforded, after flash chromatography (SiO_2, 0-100% ethyl acetate in hexanes gradient), **S3g** (77.3 mg, 69%) as an colorless oil: ^{1}H NMR (400 MHz, $CDCl_3$) δ 8.21 (d, *J* = 1.8 Hz, 1H), 7.68 (d, *J* = 8.7 Hz, 2H), 7.60 (dd, *J* = 8.7, 1.9 Hz, 1H), 7.46 (d, *J* = 8.7 Hz, 1H), 7.05 (d, *J* = 2.0 Hz, 1H), 7.02 (d, *J* = 8.1 Hz, 1H), 7.01 (d, *J* = 8.6 Hz, 2H), 6.81 (dd, *J* = 8.2, 2.0 Hz, 1H), 5.25 (s, 1H), 4.34 (m, 4H), 4.20 (m, 2H), 2.84 (s, 3H), 2.83 (s, 3H), 1.02 (s, 9H), 1.01 (s,

9H). The diastereomeric ratio (dr) of 1.1:1 was determined based on the peaks at 1.02 and 1.01 ppm.

Preparation of **2-(tert-butoxy)-2-(4-(2,3-dihydrobenzo[b][1,4] dioxin-6-yl)-2-methyl-7-(2-(trifluoromethoxy) phenyl) quinolin-3-yl) acetic acid S4g**. Applying general procedure C to **S3g** (75.00 mg, 138.47 µmoles) and ethanol (1.5 mL) in a solution of 5.0 M LiOH (0.5 mL), afforded, after prep HPLC on Xbridge™ Prep Shield Rp18, 10 µM OBD™ 19 X 250mm column (eluted with 0-100% HPLC grade submicron filtered water and Acetonitrile gradient), **S4g** (58.1 mg, 82%) as yellow solid powder: ^{1}H NMR (400 MHz, MeOD) δ 8.12 (s, 1H), 7.69 (d, *J* = 8.6 Hz, 2H), 7.57 - 7.49 (m, 2H), 7.19 (d, *J* = 1.9 Hz, 1H), 7.13 (dd, *J* = 1.9, 8.3 Hz, 1H), 7.03 (d, *J* = 8.8 Hz, 2H), 6.89 - 6.77 (m, 1H), 5.33 (s, 1H), 4.39 - 4.27 (m, 4H), 3.84 (s, 3H), 2.84 (s, 3H), 0.99 (s, 9H). The diastereomeric ratio (dr) of 1.2:1 was determined based on the peaks at 1.00 and 0.99 ppm.

Preparation of **Ethyl 2-(tert-butoxy)-2-(4-chloro-2-methyl-7-(2-(trifluoromethoxy) phenyl) quinolin-3-yl) acetate S2h**. Applying general procedure A to Ethyl 2-(7-bromo-4-chloro-2-methylquinolin-3-yl)-2-(tert-butoxy) acetate (**S1**) (102.2 mg, 61.5 µmoles), 2-trifloromethoxy phenyl boronic acid (54.7 mg, 66.4 µmoles), K_2CO_3 (101.2 mg, 186.0 µmoles), $Pd(PPh_3)_4$ (20.8 mg, 12.4 µmoles) in a solution of DMF (731 µL) and H_2O (73.1µL), afforded, after flash chromatography (SiO_2, 0-100% ethyl acetate in hexanes gradient), **S2h** (52.9 mg, 50%) as an orange oil: ^{1}H NMR (400 MHz, $CDCl_3$) δ 8.28 (d, *J* = 8.7 Hz, 1H), 8.11 (d, *J* = 1.6 Hz, 1H), 7.74 (dd, *J* = 8.7, 1.7 Hz, 1H), 7.55 (d, *J* = 2.1 Hz, 1H), 7.47 - 7.39 (m, 3H), 6.01 (s, 1H), 4.19 (dq, *J* = 11.2, 7.1 Hz, 1H), 4.19 (dq, *J* = 7.1, 1.0 Hz, 1H)2.82 (s, 3H), 1.27 (s, 9H), 1.20 (t, *J* = 7.1 Hz, 3H). The diastereomeric ratio (dr) could not be determined.

Preparation of **Ethyl 2-(tert-butoxy)-2-(4-(2,3-dihydrobenzo[b][1,4] dioxin-6-yl)-2-methyl-7-(2-(trifluoromethoxy) phenyl) quinolin-3-yl) acetate S3h**. Applying general procedure B to **S2h** (59.60 mg, 120.18 µmoles), 1,4-benzodioxane-6-boronic acid (23.79 mg, 132.20 µmoles), Cs_2CO_3 (117.47 mg, 360.54 µmoles), $Pd(OAc)_2$ (6.75 mg, 30.05 µmoles) in a solution of DMF (731 µL) and H_2O (73.1µL) afforded, after flash chromatography (SiO_2, 0-100% ethyl acetate in hexanes gradient), **S3h** (54 mg, 95%) as an orange oil: ^{1}H NMR (400 MHz, $CDCl_3$) δ 8.12 (d, *J* = 1.2 Hz, 1H), 7.56 - 7.48 (m, 3H), 7.44 - 7.37 (m, 3H), 7.08 - 6.98 (m, 2H), 6.91 - 6.83 (m, 1H), 5.27 (s, 1H), 4.39 - 4.34 (m, 4H), 4.28 - 4.14 (m, 2H), 2.85 (s,3H), 1.26 (t, *J* = 7.1 Hz, 3H), 1.04 (s, 9H). The diastereomeric ratio (dr) of 1.2:1 was determined based on the peaks at 1.04 and 1.03 ppm.

Preparation of **2-(tert-butoxy)-2-(4-(2,3-dihydrobenzo[b][1,4] dioxin-6-yl)-2-methyl-7-(2-(trifluoromethoxy) phenyl) quinolin-3-yl) acetic acid S4h**. Applying general procedure C to **S3h** (54.00 mg, 105.55 µmoles) and ethanol (123.36 µL) in a solution of 10.0 M NaOH (105.55 µL), afforded, after prep HPLC on Xbridge™ Prep Shield Rp18, 10 µM OBD™ 19 X 250mm column (eluted with 0-100% HPLC grade submicron filtered water and Acetonitrile gradient), **S4h (**31.8 mg, 62%) as white solid powder: ^{1}H NMR (400 MHz, MeOD) δ 8.10 (d, *J* = 1.1 Hz, 1H),

7.66 - 7.59 (m, 3H), 7.59 - 7.46 (m, 3H), 7.21 - 7.12 (m, 1H), 7.11 - 7.02 (m, 1H), 6.95 - 6.84 (m, 1H), 5.38 (s, 1H), 4 .47 - 4.31 (m, 4H), 2.86 (s, 3H), 1.04 (s, 9H). The diastereomeric ratio (dr) of 1.3:1 was determined based on the peaks at 1.04 and 1.03 ppm.

Preparation of **Ethyl 2-(tert-butoxy)-2-(4-chloro-7-(2-ethoxyphenyl)-2-methylquinolin-3-yl) acetate S2i**. Applying general procedure A to Ethyl 2-(7-bromo-4-chloro-2-methylquinolin-3-yl)-2-(tert-butoxy) acetate (**S1**) (100.0 mg, 241.70 µmoles), 2-ethoxy phenyl boronic acid (44.13 mg, 265.87 µmoles), Cs_2CO_3 (236.25 mg, 725.11 µmoles), $Pd(PPh_3)_4$ (22.13 mg, 60.43 µmoles) in a solution of DMF (731 µL) and H_2O (73.1µL), afforded, after flash chromatography (SiO_2, 0-100% ethyl acetate in hexanes gradient), **S2i** (50.6 mg, 54%) as an orange oil: ^{1}H NMR (400 MHz, $CDCl_3$) δ 8.21 (d, *J* = 8.7 Hz, 1H), 8.15 (s, 1H), 7.88 (d, *J* = 8.7 Hz, 1H), 7.45 (d, *J* = 7.5 Hz, 1H), 7.35 (t, *J* = 7.8 Hz, 1H), 7.08 - 7.00 (m, 2H), 6.00 (s, 1H), 4.18 (dq, *J* = 7.1, 1.0 Hz, 1H), 4.17 (dq, *J* = 7.1, 1.0 Hz, 1H) 4.08 (q, *J* = 7.0 Hz, 2H), 2.82 (s, 3H), 1.36 (t, *J* = 7.0 Hz, 3H), 1.25 (s, 9H), 1.18 (t, *J* = 7.1 Hz, 3H). The diastereomeric ratio (dr) could not be determined.

Preparation of **Ethyl 2-(tert-butoxy)-2-(4-(2,3-dihydrobenzo[b][1,4] dioxin-6-yl)-7-(2-ethoxyphenyl)-2-methylquinolin-3-yl) acetate S3i**. Applying general procedure B to **S2i** (50.6 mg, 116.44 µmoles), 1,4-benzodioxane -6- boronic acid (23.1 mg, 128.08 µmoles), Cs_2CO_3 (113.81 mg, 128.08 µmoles), $Pd(OAc)_2$ (6.54 mg, 29.11 µmoles) in a solution of DMF (731 µL) and H_2O (73.1µL), afforded, after flash chromatography (SiO_2, 0-100% ethyl acetate in hexanes gradient), **S3h** (32.8 mg, 52%) as an orange oil: ^{1}H NMR (400 MHz, $CDCl_3$) δ 8.17 (d, *J* =1.6 Hz, 1H), 7.65 (dd, *J* = 8.7, 1.7 Hz, 1H), 7.48 - 7.40 (m, 2H), 7.36 - 7.30 (m, 1H), 7.08 - 6.99 (m, 4H), 6.92 - 6.83 (m, 1H), 5.26 (s, 1H), 4.38 - 4.32 (m, 4H), 4.27 - 4.13 (m, 2H), 4.07 (q, *J* = 7.0 Hz, 2H), 2.84 (s, 3H), 1.34 (t, *J* = 7.0 Hz, 3H), 1.25 (t, *J* = 7.0 Hz, 3H), 1.02 (s, 9H). The diastereomeric ratio (dr) of 1.2:1 was determined based on the peaks at 1.03 and 1.02 ppm.

Preparation of **2-(tert-butoxy)-2-(4-(2,3-dihydrobenzo[b][1,4] dioxin-6-yl)-7-(2-ethoxyphenyl)-2-methylquinolin-3-yl) acetic acid S4i**. Applying general procedure C to**S3i** (32.8 mg, 59.03 µmoles) and ethanol (1.0 mL) in a solution of 10.0 M NaOH (500.0 µL), afforded, after prep HPLC on Xbridge™ Prep Shield Rp18, 10 µM OBD™ 19 X 250mm column (eluted with 0-100% HPLC grade submicron filtered water and Acetonitrile gradient), **S4i (**31.8 mg, 95%) as white solid powder: ^{1}H NMR (400 MHz, MeOD) δ ^{1}H NMR (400 MHz, MeOD) 8.13 (t, *J* = 1.8 Hz, 1H), 7.69 - 7.64 (m, 1H), 7.50 (q, *J* = 6.8 Hz, 1H), 7.43 - 7.33 (m, 2H), 7.20 - 7.11 (m, 1H), 7.10 - 7.03 (m, 3H), 6.89 - 6.81 (m, 1H), 5.34 (s, 1H), 4.41 - 4.28 (m, 4H), 4.07 (q, *J* = 7.2 Hz, 2H), 2.86 (s, 3H), 1.30 (q, *J* = 7.0 Hz, 3H), 1.01 (s, 9H), 1.00 (s, 9H). The diastereomeric ratio (dr) of 1.3:1 was determined based on the peaks at 0.997 and 0.995 ppm.
